# Calcium-Dependent and Synapsin-Dependent Pathways for the Presynaptic Actions of BDNF

**DOI:** 10.3389/fncel.2017.00075

**Published:** 2017-03-24

**Authors:** Qing Cheng, Sang-Ho Song, George J. Augustine

**Affiliations:** ^1^Department of Neurobiology, Duke University Medical CenterDurham, NC, USA; ^2^Center for Functional Connectomics, Korea Institute of Science and TechnologySeoul, South Korea; ^3^Lee Kong Chian School of Medicine, Nanyang Technological UniversitySingapore, Singapore; ^4^Institute of Molecular and Cell BiologySingapore, Singapore

**Keywords:** neurotrophins, synapsins, neurotransmitter release, TRP channels

## Abstract

We used cultured hippocampal neurons to determine the signaling pathways mediating brain-derived neurotrophic factor (BDNF) regulation of spontaneous glutamate and GABA release. BDNF treatment elevated calcium concentration in presynaptic terminals; this calcium signal reached a peak within 1 min and declined in the sustained presence of BDNF. This BDNF-induced transient rise in presynaptic calcium was reduced by SKF96365, indicating that BDNF causes presynaptic calcium influx via TRPC channels. BDNF treatment increased the frequency of miniature excitatory postsynaptic currents (mEPSCs). This response consisted of two components: a transient component that peaked within 1 min of initiating BDNF application and a second component that was sustained, at a lower mEPSC frequency, for the duration of BDNF application. The initial transient component was greatly reduced by removing external calcium or by treatment with SKF96365, as well as by Pyr3, a selective blocker of TRPC3 channels. In contrast, the sustained component was unaffected in these conditions but was eliminated by U0126, an inhibitor of the MAP kinase (MAPK) pathway, as well as by genetic deletion of synapsins in neurons from a synapsin triple knock-out (TKO) mouse. Thus, two pathways mediate the ability of BDNF to enhance spontaneous glutamate release: the transient component arises from calcium influx through TRPC3 channels, while the sustained component is mediated by MAPK phosphorylation of synapsins. We also examined the ability of these two BDNF-dependent pathways to regulate spontaneous release of the inhibitory neurotransmitter, GABA. BDNF had no effect on the frequency of spontaneous miniature inhibitory postsynaptic currents (mIPSCs) in neurons from wild-type (WT) mice, but surprisingly did increase mIPSC frequency in synapsin TKO mice. This covert BDNF response was blocked by removal of external calcium or by treatment with SKF96365 or Pyr3, indicating that it results from calcium influx mediated by TRPC3 channels. Thus, the BDNF-activated calcium signaling pathway can also enhance spontaneous GABA release, though this effect is suppressed by synapsins under normal physiological conditions.

## Introduction

The neurotrophin brain-derived neurotrophic factor (BDNF) plays multiple roles in neuronal development, synapse maturation and synaptic plasticity (Park and Poo, 2013). In addition to its long-term developmental effects, BDNF acutely affects synaptic transmission and other neuronal physiological properties (Lessmann et al., 1994; Kang and Schuman, 1995; Levine et al., 1995; Gottschalk et al., 1998; Li et al., 1998; Mizoguchi et al., 2003; Amaral and Pozzo-Miller, 2007a; McGurk et al., 2011). The most prominent acute effect of BDNF is to increase the frequency of miniature excitatory postsynaptic currents (mEPSCs; Li et al., 1998; Taniguchi et al., 2000; Tyler and Pozzo-Miller, 2001), indicating an enhancement of spontaneous glutamate release from presynaptic terminals. In addition, BNDF has been shown to variably regulate the presynaptic GABA release, either having no effect (Shinoda et al., 2014; Colino-Oliveira et al., 2016), increasing (Bolton et al., 2000; Wardle and Poo, 2003), or decreasing (Brünig et al., 2001) the frequency of spontaneous miniature inhibitory postsynaptic currents (mIPSCs).

The signaling mechanisms underlying the presynaptic actions of BDNF remain elusive. Upon binding to trkB receptors, BDNF activates multiple downstream signaling molecules, including phospholipase C (PLC), MAP kinase (MAPK) and PI3K (Reichardt, 2006). In turn, the substrates of these kinases are largely responsible for carrying out the signaling actions of BDNF. Several studies have reported that BDNF increases intracellular calcium levels, which could underlie the enhancement of spontaneous glutamate release (Li et al., 1998; Boulanger and Poo, 1999; Pozzo-Miller et al., 1999). Such a rise in presynaptic calcium levels could be due to the activation of the PLC pathway (Reichardt, 2006), which in turn could activate calcium influx mediated by TRPC channels (Amaral and Pozzo-Miller, 2007b) and/or release of calcium from intracellular stores (Amaral and Pozzo-Miller, 2012).

The presynaptic actions of BDNF also may involve synapsins, a family of proteins that associate with synaptic vesicles and regulate the release of various neurotransmitters (Hilfiker et al., 1999; Gitler et al., 2004, 2008; Cesca et al., 2010; Kile et al., 2010; Song and Augustine, 2016). Synapsins are encoded by three mammalian genes, which generate multiple isoforms through alternative splicing. Deletion of the genes for either synapsin I or synapsin II (Jovanovic et al., 2000), as well as all three synapsins (Kao et al., 2017), attenuates the presynaptic response to BDNF, implicating synapsins in BDNF enhancement of glutamate release. Each synapsin isoform contains sites for phosphorylation by several protein kinases, including MAPK (Hilfiker et al., 2005), and these sites have been implicated as downstream substrates for MAPK phosphorylation during BDNF signaling (Jovanovic et al., 2000).

To differentiate the roles of synapsins and calcium in the presynaptic actions of BDNF, we employed synapsin triple-knockout (TKO) mice (Gitler et al., 2004), ion substitution and pharmacological approaches. We identified two parallel pathways, involving both TRPC-channel mediated calcium influx and MAPK-mediated synapsin phosphorylation, that make kinetically distinct contributions to BDNF enhancement of spontaneous glutamate release. The TRPC-mediated pathway also enhances spontaneous release of GABA, though this effect is normally inhibited by synapsins under physiological conditions.

## Materials and Methods

### Hippocampal Neuronal Cultures

Homozygous synapsin TKO mice and matching triple wild-type (TWT) mice were produced as described previously (Feng et al., 2002; Gitler et al., 2004). The procedures used to maintain and use these mice were approved by the Animal Care and Use Committees of Duke University and the Biopolis Biological Resource Center. Newborn pups (postnatal day 0–1) were used to prepare hippocampal neurons. Microisland cultures of hippocampal neurons were prepared as described in Bekkers and Stevens (1991), with the addition of glia feeder cells to support neuronal survival. Neurons were allowed to mature for 10–14 days before being used for electrophysiological recordings.

In other experiments (calcium imaging, measurements of spontaneous miniature IPSCs), high-density cultures of hippocampal neurons (e.g., Bi and Poo, 1998) were prepared and examined 10–18 days later.

### Electrophysiological Acquisition and Data Analysis

For recording spontaneous mEPSCs, whole-cell patch-clamp recordings were made from single neurons on microislands. Patch pipettes (4–6 MΩ) were filled with intracellular solution containing (in mM): 50 K-glutamate, 71 K-gluconate (Fluka, Buchs, Switzerland), 15 NaCl, 6 MgCl_2_, 0.5 EGTA, 5 Na_2_ATP, 0.3 Na_2_GTP and 20 HEPES-KOH, pH 7.3 (285 mOsm). The extracellular solution contained (in mM): 150 NaCl, 3 KCl, 2 CaCl_2_, 2 MgCl_2_, 20 glucose and 10 HEPES-NaOH, pH 7.3 (310 mOsm). All materials were from Sigma, unless specified otherwise. A HEKA EPC-9D amplifier (HEKA, Lambrecht/Pfalz, Germany) was used to voltage clamp neurons at a holding potential of −70 mV. Under these conditions, spontaneous EPCSs are solely due to mEPSCs that were blocked by the AMPA receptor antagonist, CNQX (20 μM). Spontaneous synaptic events were semi-automatically analyzed using the MiniAnalysis program (Synaptosoft, Decatur, GA, USA). Values were compared by the Student’s *t* test, with error bars shown in the figures indicating the SEM. BDNF was purchased from Peprotech (Rock Hill, NJ, USA) and BDNF-containing external solution was delivered via air pressure (3–5 psi; Picospritzer, General Valve, Firfield, NJ, USA) from a glass pipette (tip diameter 30–50 μm) placed 100 μm away.

Similar procedures were used to record spontaneous miniature IPSCs, the intracellular solution contained (in mM): 140 CsCl, 4 NaCl, 0.5 CaCl_2_, 5 EGTA, 2 MgATP, 0.4 Na_3_GTP, 10 HEPES-KOH and 10 QX-314 (pH 7.4, adjusted with CsOH). The holding potential for mIPSC recordings was also −70 mV. mIPSCs were recorded in the presence of tetrodotoxin (1 μM), APV (50 μM) and CNQX (20 μM) to eliminate action potentials and excitatory postsynaptic currents. Under these conditions, the responses must be mIPSCs. All recordings were made at room temperature (21–25°C).

### Calcium Imaging

To identify presynaptic terminals, neurons were transfected with a synaptophysin-mCherry plasmid (a generous gift from Dr. M. Kennedy, Duke University) via lipofectamine transfection at day 4–6, with neurons imaged at days 10–14. The calcium indicator dye Fluo4-AM (1 μM; Invitrogen, CA, USA) was then loaded into the neurons at 37°C for 30 min. Neurons were imaged after 30 additional minutes of incubation in dye-free solution. In these experiments, the external solution contained 10 μM CNQX, 25 μM APV and 1 μM TTX to prevent calcium signals associated with glutamate receptors or action potentials. Images of identified presynaptic terminals were acquired before and during 5 min application of BDNF. Presynaptic terminals were identified as structures with punctate synaptophysin-mCherry fluorescence signals <2 μm^2^ in area. Images were viewed through a 40×, 0.7 NA objective of an inverted epifluorescence microscope (IX70, Olympus) equipped with a mercury lamp; dye excitation was controlled by a mechanical shutter (Uniblitz). The optical filters used for imaging Fluo4 were 470 ± 20 nm (excitation), 505 nm (dichroic mirror), and 530 ± 20 nm (emission); for mCherry these were 540 ± 10 nm (excitation), 570 nm (dichroic mirror), and 590 nm long pass (emission). All filters were obtained from Chroma Technologies, Rockingham, VT, USA. Images were obtained with a back-illuminated, cooled CCD camera with the on-chip multiplication gain control (Cascade 512B, Photometrics, Tucson, AZ, USA). Images were acquired and analyzed by RatioTool software (ISee Imaging Systems, Raleigh, NC, USA). Fluo4 measurements were made from 15–20 presynaptic boutons in each experiment.

## Results

We used cultured hippocampal neurons to examine the signaling pathways underlying the acute presynaptic actions of BDNF.

### BDNF-Mediated Calcium Influx via TRPC Channels

**Figure 1:**
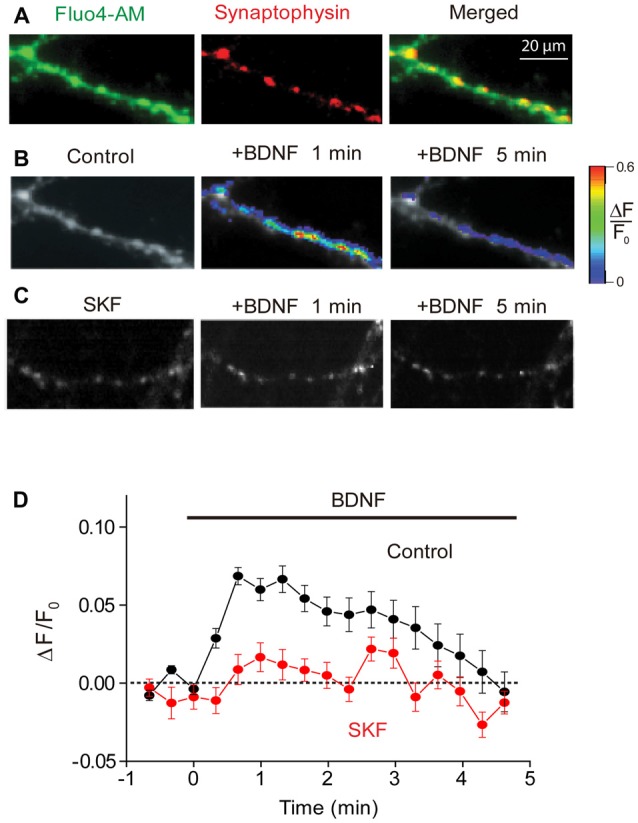
**Brain-derived neurotrophic factor (BDNF) induced calcium rise at presynaptic terminals.** **(A)** Representative images of presynaptic terminals from triple wild-type (TWT) neurons transfected with synaptophysin-mCherry (red) after Fluo4-AM loading (green). Images of Fluo4 fluorescence during 1st (left) and 5th minute (right) of BDNF application in the absence **(B)** and presence of SKF96365 **(C)**. Scale at right indicates relative changes in Fluo4 fluorescence relative to baseline levels measured prior to BDNF treatment (ΔF/F_0_). **(D)** Time course of BDNF-induced rise in presynaptic calcium, measured in control conditions (black) and in the presence of SKF96365 (red).

Although several studies have demonstrated a BDNF-induced rise in intracellular calcium concentration ([Ca^2+^]_i_), these measurements were made from neuronal cell bodies or dendritic spines (Li et al., 1998; Amaral and Pozzo-Miller, 2007b, 2012). To determine whether presynaptic [Ca^2+^]_i_ levels are also affected by BDNF, we began by using fluorescence imaging to directly monitor [Ca^2+^]_i_ in presynaptic terminals. To identify these terminals, we transfected neurons with a plasmid encoding a synaptic vesicle protein, synaptophysin, fused to the red fluorescent protein mCherry. After transfection for 5–6 days, synaptophysin-mCherry distributed in a punctuate pattern consistent with localization of its native counterpart to presynaptic terminals (Kennedy and Ehlers, 2011). We then used a low-affinity fluorescent calcium indicator dye, Fluo4-AM, to measure [Ca^2+^]_i_ changes in such synaptophysin-mCherry labeled terminals (Figure 1A).

Presynaptic [Ca^2+^]_i_, as indicated by Fluo4 fluorescence, increased upon application of BDNF (Figure 1B). The BDNF-induced rise in [Ca^2+^]_i_ was modest in amplitude (Figure 1D), with the peak change of 0.07 ± 0.02 (*n* = 7), corresponding to a volume-averaged [Ca^2+^]_i_ rise of approximately 100 nM. This rise in [Ca^2+^]_i_ was transient, reaching a peak within 30 s and then slowly returning to baseline levels by the end of a 5-min long application of BDNF (Figure 1D). This indicates that BDNF does indeed elevate [Ca^2+^]_i_ in presynaptic terminals.

Because calcium influx through TRPC3 channels has been implicated in the actions of BDNF (Amaral and Pozzo-Miller, 2007b, 2012), and these channels co-localize with several presynaptic markers (Singh et al., 2004), we next asked whether the BDNF-mediated rise in presynaptic [Ca^2+^]_i_ is mediated by TRPC channels. We examined the actions of SKF96365, a blocker of store-operated cation channels including TRPC channels (Merritt et al., 1990; Zhu et al., 1998), on the BDNF-induced rise in presynaptic [Ca^2+^]_i_. Pretreatment of neurons with SKF96365 greatly reduced the rise in presynaptic [Ca^2+^]_i_ caused by BDNF (Figures 1C,D). This drug reduced the peak change in Fluo4 signal caused by BDNF from 0.07 ± 0.02 to 0.02 ± 0.01 (*n* = 6; *p* < 0.01, Student’s *t*-test), while the fluorescence signal integrated over the first 120 s of BDNF application was reduced from 8.2 ± 2.4 ΔF/F.s in control conditions to 0.6 ± 0.4 ΔF/F.s in the presence of SKF96365 (*p* < 0.03, Student’s *t*-test). This indicates that the majority of the presynaptic [Ca^2+^]_i_ signal associated with BDNF is due to Ca^2+^ influx through TRPC or a similar store-operated cation channel.

### Role of Calcium Influx in BDNF Enhancement of Glutamate Release

**Figure 2:**
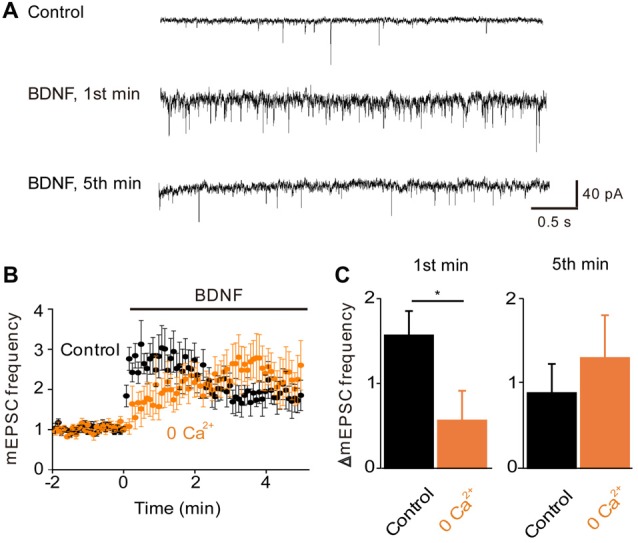
**Role of calcium influx in BDNF enhancement of spontaneous glutamate release.** **(A)** Representative traces of miniature EPSCs before and during BDNF application from a WT autaptic neuron. **(B)** Time course of changes in normalized miniature excitatory postsynaptic current (mEPSC) frequency during BDNF application (bar) in normal external solution (black) and calcium-free external solution (orange, *n* = 6). **(C)** Changes in mEPSC frequency during 1st (left) and 5th (right) minute of BDNF application. Throughout the article, values indicate mean *±* standard error of the mean and *denotes a significant difference (*p* < 0.05).

To examine the role of TRPC-mediated Ca^2+^ influx in the regulation of spontaneous glutamate release by BDNF (Lessmann et al., 1994; Li et al., 1998), we measured mEPSCs in microisland-cultured hippocampal neurons. Treatment of these neurons with BDNF increased the frequency of mEPSCs (Figure 2A). This response consisted of two kinetically-distinct components: an initial, transient rise that reached a peak within 1 min and a secondary, sustained increase in mEPSC frequency that was sustained throughout the entire 5-min long application of BDNF (Figure 2B, black symbols). To quantify the effect of BDNF, we measured the relative increase in mEPSC frequency during the 1st minute of BDNF application, representing the transient initial component, and during the 5th minute of BDNF application, to assess the sustained component (Figure 2C, black). The increase in mEPSC frequency occurring during the first minute of BDNF application was 157 ± 29% (mean ± SEM) and was approximately half as large (89 ± 34%) after 5 min of BDNF treatment. In addition to this acute presynaptic action of BDNF, we asked whether BDNF exerts a postsynaptic effect by measuring the amplitude of mEPSCs. We found that the amplitude of mEPSCs did not change significantly during a 5-min long application of BDNF; mean mEPSC amplitude was 25.8 ± 2.6 pA in control conditions and 25.6 ± 2.2 pA in the presence of BDNF. Therefore, though BDNF is known to increase mEPSC amplitude when applied for longer times (Levine et al., 1998), our data indicate that the initial locus of action of BDNF at glutamatergic synapses is presynaptic under our conditions.

**Figure 3:**
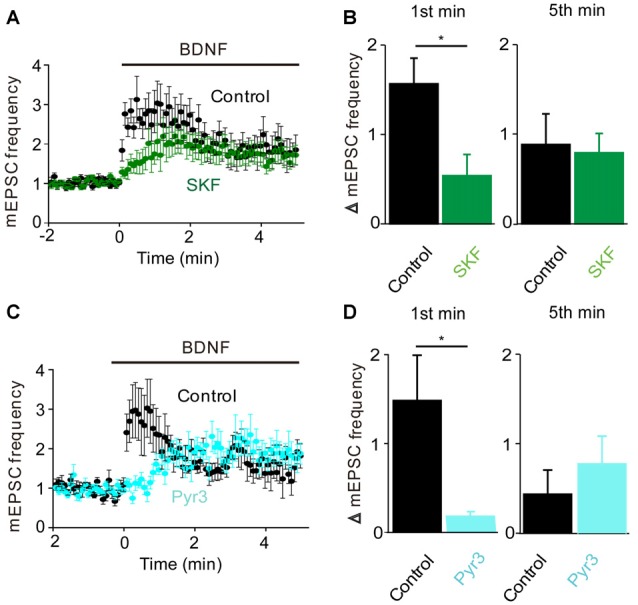
**TRPC3 mediates spontaneous glutamate release by BDNF.** **(A)** Changes in mEPSC frequency caused by BDNF application in the absence (black, *n* = 13) and presence of SKF95365 (green, *n* = 6). **(B)** Changes in mEPSC frequency during 1st (left) and 5th (right) minute of BDNF application in control conditions and during treatment with SKF96365. **(C)** Changes in mEPSC frequency caused by BDNF application in the absence (black, *n* = 5) and presence of Pyr3 (sky blue, *n* = 5). **(D)** Changes in mEPSC frequency during 1st (left) and 5th (right) minute of BDNF application in control conditions and during treatment with Pyr3.

To determine whether the BDNF-induced Ca^2+^ influx shown in Figure 1 is involved in the increase in mEPSC frequency, we examined the actions of BDNF in a calcium-free external solution containing the Ca^2+^ chelator EGTA (Figure 2B, orange). In this condition, the initial BDNF-induced increase in mEPSC frequency was reduced significantly to 33% of control (*n* = 8; *p* < 0.03, Student’s *t*-test), while the secondary sustained increase in mEPSC frequency was unaffected (135% of control, *p* = 0.43, Figure 2C). This indicates that the initial component of the BDNF response at least partially requires Ca^2+^ influx, while the sustained component does not. Similar to the Ca^2+^ influx induced by BDNF, the initial BDNF-induced increase in mEPSC frequency was reduced to 33% of control (*n* = 8, *p* = 0.02) by SKF96365 (Figures 3A,B, green). In contrast, the sustained component was not significantly affected by SKF96365 (89% of control, *p* = 0.67; Figure 3B). Furthermore, Pyr3, a more specific blocker of TRPC3 channels (Kiyonaka et al., 2009) almost entirely eliminated the initial BDNF-induced increase in mEPSC frequency (12% of control, *n* = 5, *p* = 0.03) but spared the sustained component (178% of control, *n* = 5, *p* = 0.43; Figures 3C,D, cyan). In sum, it appears that the initial presynaptic response to BDNF involves Ca^2+^ influx via TRPC3 channels, while the sustained component does not.

### Role of MAPK Pathway

**Figure 4:**
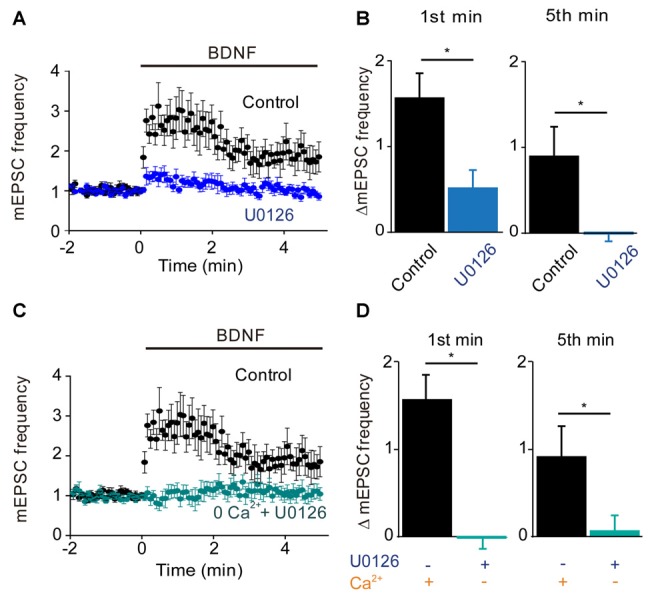
**MAP kinase (MAPK)-dependent and calcium-dependent responses to BDNF. (A)** Time course of changes in normalized mEPSC frequency during BDNF application in the absence and presence of the MAPK inhibitor, U0126 (20 μM). U0126 was bath perfused 5 min prior BDNF application. **(B)** Changes in mEPSC frequency during 1st (left) and 5th (right) minute of BDNF application, with and without U0126. **(C)** Time course of changes in normalized mEPSC frequency in neurons in normal external solution (black) or in calcium-free solution containing EGTA and U0126 (aqua). **(D)** Changes in mEPSC frequency during 1st (left) and 5th minute (right) of BDNF application in the indicated conditions.

Given that the MAPK pathway has been implicated in the presynaptic actions of BDNF (Jovanovic et al., 1996), we next determined the role of this kinase by examining the effects of the MAPK inhibitor U0126. Treatment with this compound completely prevented the sustained enhancement of mEPSC frequency by BDNF (−3% of control, *n* = 9, *p* = 0.001), while also reducing the transient initial component of the BDNF response (Figure 4A). The residual increase in mEPSC frequency (17% of control) was transient, returning to baseline during the first 2 min of BDNF treatment (Figure 4A). Quantification revealed the absence of a sustained response and a reduction in the initial response to BDNF in the presence of U0126 (Figure 4B, blue). Thus, the MAPK pathway seems to be responsible for the sustained response to BDNF but not for the transient, initial response.

Taken together, our results suggest that TRPC3-mediated Ca^2+^ influx is involved in the transient, initial component of the BDNF response while the MAPK pathway is involved in the late, sustained component. If this is the case, then blocking both Ca^2+^ influx and MAPK activity should completely abolish the ability of BDNF to enhance spontaneous glutamate release. Indeed, when neurons were treated with U0126 in Ca^2+^-free external solution, BDNF failed to increase mEPSC frequency (Figure 4C). Both the transient initial component (−1% of control, *n* = 6, *p* = 0.01, Figure 4D, aqua) and the sustained response to BDNF (5% of control, *n* = 6, *p* = 0.001, Figure 4D, aqua) were absent in these conditions. We therefore conclude that Ca^2+^ influx via TRPC3 channels mediates the transient, initial BDNF response while MAPK mediates the late, sustained response. Further, we can conclude that these two signaling pathways together are completely responsible for the presynaptic actions of BDNF under our conditions.

### Synapsin Involvement in MAPK Pathway

**Figure 5:**
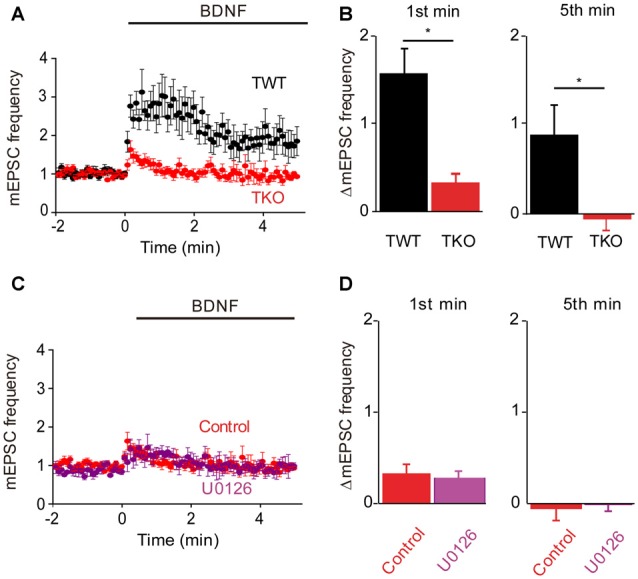
**Role of synapsins in MAPK pathway.** **(A)** Time course of changes in normalized mEPSC frequency during BDNF application for TWT (black, *n* = 13) and synapsin triple knock-out (TKO) neurons (red, *n* = 12). **(B)** Changes in mEPSC frequency during 1st (left) and 5th minute (right) of BDNF application in TWT and TKO neurons. **(C)** Time course of changes in normalized mEPSC frequency during BDNF application in TKO neurons in the absence (red) or presence (purple) of U0126.** (D)** Changes in mEPSC frequency during 1st (left) and 5th minute (right) of BDNF application in TKO neurons with and without U0126 treatment.

Having identified the kinetic components associated with Ca^2+^ influx and MAPK activation, we next could assess the contribution of synapsins as potential MAPK substrates. For this purpose, we cultured neurons from synapsin TKO mice (Gitler et al., 2004) and examined their responses to BDNF. The dual-pathway model we have proposed predicts that TKO neurons would exhibit the transient, initial response to BDNF but not the late, sustained response. The BDNF response of TKO neurons was greatly attenuated (Figure 5A). Indeed, as predicted the sustained BDNF response was completely abolished (−5% of control, *n* = 12, *p* < 0.001), while the transient response was reduced but preserved (20% of control, *n* = 12, *p* = 0.01, Figure 5B, red). The residual transient response to BDNF observed in the TKO neurons was remarkably similar, both in magnitude and in time course, to the response of WT neurons after blockade of the MAPK pathway (compare to Figure 4A). This is consistent with the hypothesis that synapsins are the main substrates of MAPK in mediating the sustained response to BDNF. Further support for this notion come from experiments where TKO neurons were treated with U0126: under these conditions, U0126 caused no change in the BDNF response (Figure 5C). Neither the initial, transient component (84% of control, *n* = 5, *p* = 0.19) nor the sustained component 67% of control, *p* = 0.28) of TKO neurons were affected by U0126 treatment (Figure 5D). This occlusion of the effect of the MAPK inhibitor strongly implicates synapsins as the major substrates for MAPK in mediating the sustained presynaptic response to BDNF.

**Figure 6:**
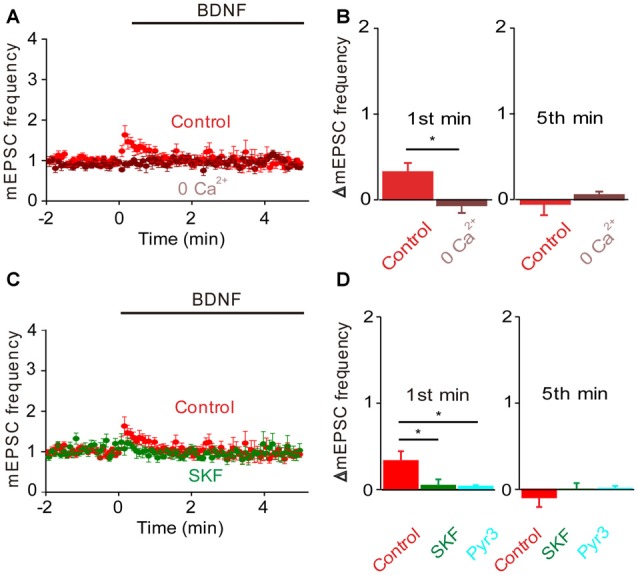
**Role of calcium influx in the BDNF response of synapsin TKO neurons.** Either removal of calcium **(A)** or inhibiting TRPC channels **(C)** abolished the residual increase in mEPSC frequency produced in TKO neurons by BDNF. Data from TKO neurons in normal (red, *n* = 12) and calcium-free external solution (brown) shown in **(A)**; data from TKO neurons in the absence (red) and presence of SKF95365 (green, *n* = 6) shown in **(C)**. **(B)** Changes in mEPSC frequency during 1st (left) and 5th minute (right) of BDNF application in TKO neurons in normal and calcium free solution. **(D)** Changes in mEPSC frequency during 1st (left) and 5th minute (right) of BDNF application in TKO neurons in the absence or presence of SKF95365 and Pyr3 (sky blue, *n* = 6).

**Figure 7:**
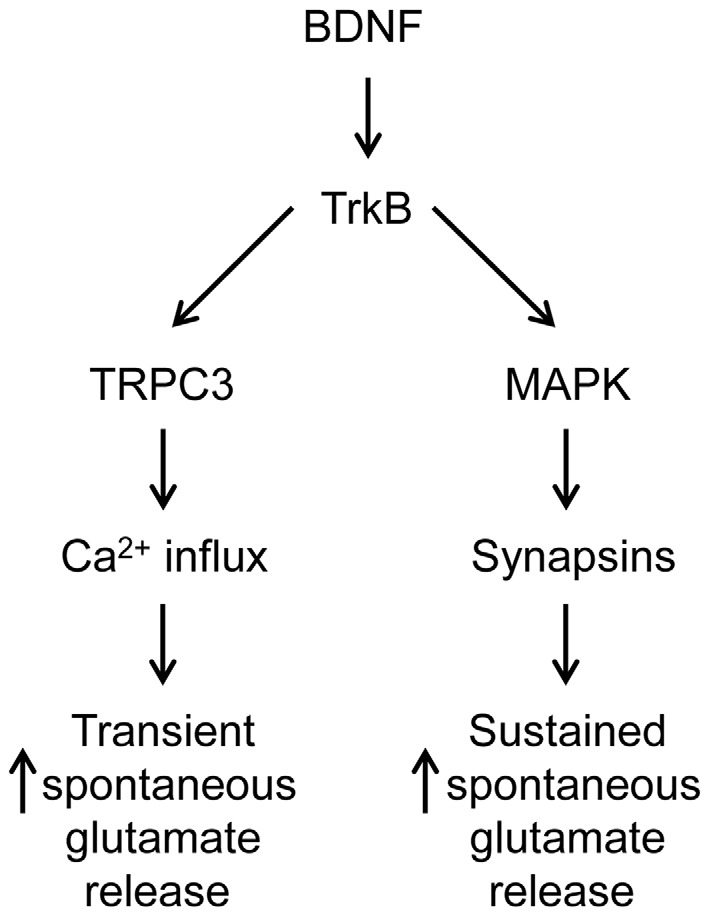
**Dual signaling pathway model for BDNF action at glutamatergic synapses**.

In TKO neurons there was a residual initial component of BDNF response both in the presence and the absence of MAPK activity. According to our model, this component is mediated by the BDNF-activated Ca^2+^ influx pathway. We tested this hypothesis by treating TKO neurons with Ca^2+^-free external solution. In such conditions, the BDNF-induced transient increase in mEPSC frequency was completely eliminated (−20% of control, *n* = 7, *p* = 0.02, Figures 6A,B, brown). This indicates that the initial, transient increase in mEPSC frequency of TKO neurons requires an influx of external Ca^2+^. Further, this component was abolished following blockade of TRPC channels by SKF96365 (13% of control, *n* = 6, *p* = 0.048; *t*-test, analyses of variance (ANOVA) followed by Tukey’s *post hoc* test, *p* = 0.03, Figures 6C,D, green) and blockade of TRPC3 channels by Pyr3 (10% of control, *n* = 6, *p* = 0.02, *t*-test; ANOVA followed by Tukey’s *post hoc* test, *p* = 0.025, Figures 6C,D, cyan). Thus, the synapsin-independent transient response to BDNF requires Ca^2+^ influx through TRPC3 channels. We therefore conclude that the presynaptic actions of BDNF can be explained by two parallel signaling pathways: the initial, transient response is mediated by influx of external Ca^2+^ through TRPC3 channels, while the slower, persistent response is mediated by MAPK phosphorylation of synapsins. These conclusions are summarized in Figure 7.

### BDNF Regulation of GABA Release

**Figure 8:**
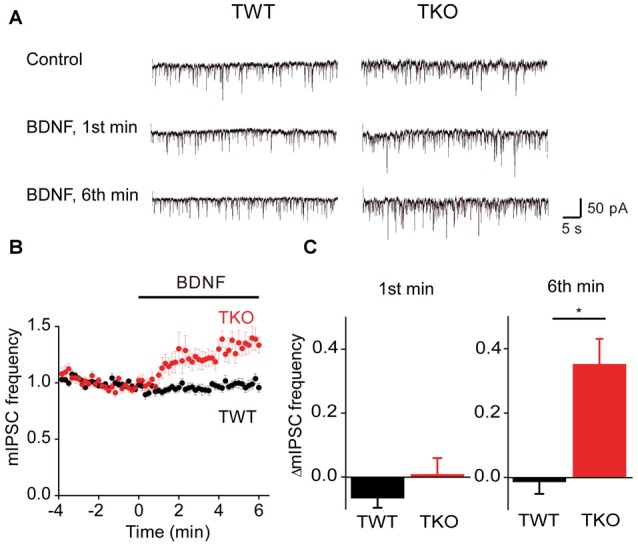
**BDNF regulation of spontaneous GABA release.** **(A)** Representative miniature IPSCs before and during BDNF application, recorded in TWT neurons (left) and TKO neurons (right). **(B)** Time course of changes in normalized miniature inhibitory postsynaptic current (mIPSC) frequency during BDNF application for TWT (black, *n* = 21) and synapsin TKO neurons (red, *n* = 18). **(C)** Changes in mIPSC frequency during 1st (left) and 6th minute (right) of BDNF application.

BDNF reportedly has variable acute effects on GABA release (Frerking et al., 1998; Bolton et al., 2000; Wardle and Poo, 2003; Canas et al., 2004; Shinoda et al., 2014). We revisited the acute actions of BDNF on spontaneous GABA release, specifically to determine whether the model for presynaptic signaling we developed for acute BDNF regulation of spontaneous glutamate release (Figure 7) is applicable to BDNF regulation of GABA release. For this purpose, miniature IPSCs (mIPSCs) were recorded in high-density cultures of hippocampal neurons and BDNF was applied for 6 min. In TWT neurons, mIPSC frequency was unaffected by BDNF (Figures 8A–C). Similarly, BDNF did not affect mIPSC amplitude, which was 29.6 ± 1.7 pA in control conditions and 29.9 ± 1.7 pA in the presence of BDNF. These results are consistent with previous reports that application of BDNF for 5–6 min does not affect mIPSC frequency or amplitude (Shinoda et al., 2014; Colino-Oliveira et al., 2016). However, in synapsin TKO neurons, BDNF application increased mIPSC frequency (Figure 8A). The time course of this GABAergic presynaptic response to BDNF was slower than that of spontaneous glutamate release, with mIPSC frequency gradually increasing over the entire time of BDNF application (Figure 8B). As a result, there was no increase in mIPSC frequency during the 1st minute of BDNF application (Figure 8C) but a significant increase in mIPSC frequency in TKO neurons (36% of TWT; *n* = 18) in comparison to TWT neurons (−1 ± 3% change; *n* = 21) after 6 min of BDNF treatment. Because there was no initial increase in mIPSC frequency, in subsequent figures we measured only the increase in mIPSC during the final minute of the 6-min long application of BDNF. In TWT neurons, BDNF caused no significant change in mIPSC amplitude, which was 28.5 ± 0.9 pA in control conditions and 28.7 ± 0.9 pA in the presence of BDNF. These results indicate that BDNF has a covert ability to regulate spontaneous GABA release that is observed after genetic deletion of synapsins. Thus, spontaneous glutamate and GABA release are differentially regulated by BDNF: synapsins play a positive role in BDNF signaling in glutamatergic terminals and a negative role in GABAergic terminals.

**Figure 9:**
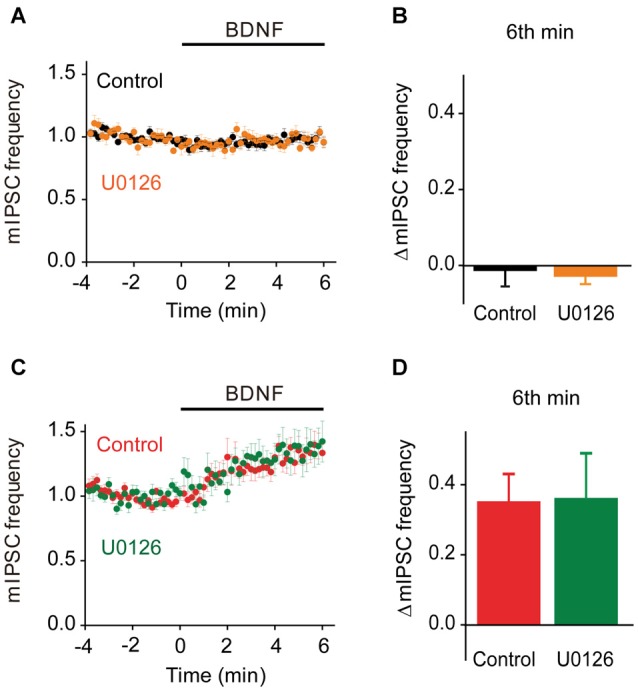
**MAPK-independent BDNF effects on spontaneous GABA release.** **(A)** Time course of changes in normalized mIPSC frequency during BDNF application for TWT neurons without (black, *n* = 21) and with (orange, *n* = 10) MAPK inhibitor U0126 (20 μM). **(B)** Changes in mIPSC frequency during 6th minute of BDNF application in TWT neurons, with and without U0126 treatment. **(C)** Time course of changes in normalized mIPSC frequency in TKO neurons during BDNF application without (red, *n* = 18) and with (green, *n* = 10) U0126 treatment. **(D)** Changes in mIPSC frequency in TKO neurons during 6th minute of BDNF application, with and without U0126.

To evaluate the role of the MAPK pathway in regulation of spontaneous GABA release, we examined the effect of U0126 on mIPSC frequency during BDNF application. In TWT neurons, BDNF had no effect on mIPSC frequency in the presence of the MAPK inhibitor (Figures 9A,B). Thus, even though synapsins appear to inhibit BDNF signaling in these neurons, blockade of MAPK signaling does not affect this inhibition. Similarly, the BDNF-induced increase in mIPSC frequency observed in TKO neurons was unaffected, in time course (Figure 9C) or in magnitude (Figure 9D), by the MAPK inhibitor. This indicates that the synapsin-independent increase in spontaneous GABA release observed in TKO neurons also is not regulated by the MAPK pathway.

**Figure 10:**
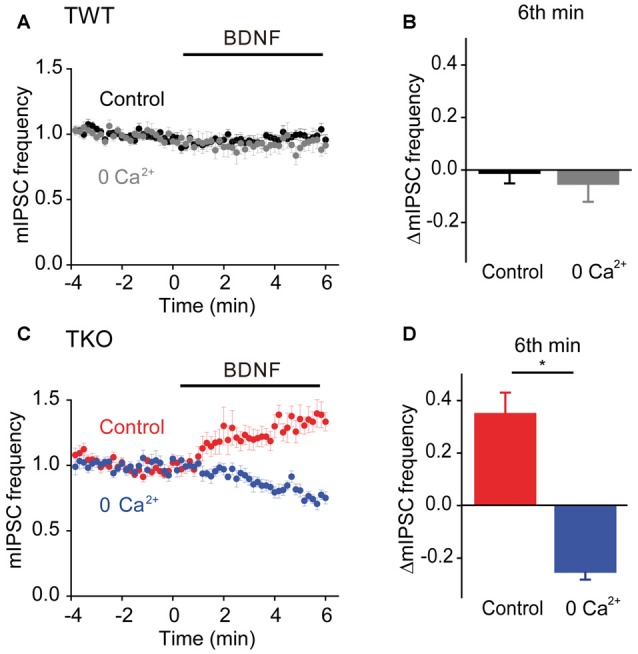
**Role of calcium influx in presynaptic action of BDNF on spontaneous GABA release in TKO neurons.** **(A)** Time course of changes in normalized mIPSC frequency during BDNF application for TWT neurons in normal (black, *n* = 21) and calcium-free gray, *n* = 14) external solution. **(B)** Changes in mIPSC frequency in TWT neurons during 6th minute of BDNF application in normal and calcium-free external solution. **(C)** Time course of changes in normalized mIPSC frequency in TKO neurons during BDNF application in normal (red, *n* = 18) and calcium-free (blue, *n* = 13) external solution. **(D)** Changes in mIPSC frequency in TKO neurons during 6th minute of BDNF application, in the absence and presence of external calcium.

Given the role of MAPK- and synapsin-independent Ca^2+^ influx in BDNF regulation of spontaneous glutamate release (Figure 7), we hypothesized that such influx might also mediate the BDNF response of TKO neurons. To test this hypothesis, we examined the effect of BDNF in Ca^2+^-free external solution. In TWT neurons, mIPSC frequency was unaffected by BDNF in Ca^2+^-free conditions (Figures 10A,B). However, the BDNF-induced increase in mIPSC frequency observed in TKO neurons was completely abolished (Figure 10C). Instead, BDNF caused a mild decrease in mIPSC frequency in these conditions (Figure 10D). This indicates that BDNF increases spontaneous GABA release in TKO neurons by causing Ca^2+^ influx, similar to the case for the initial component of the BDNF-mediated increase in spontaneous glutamate release.

**Figure 11:**
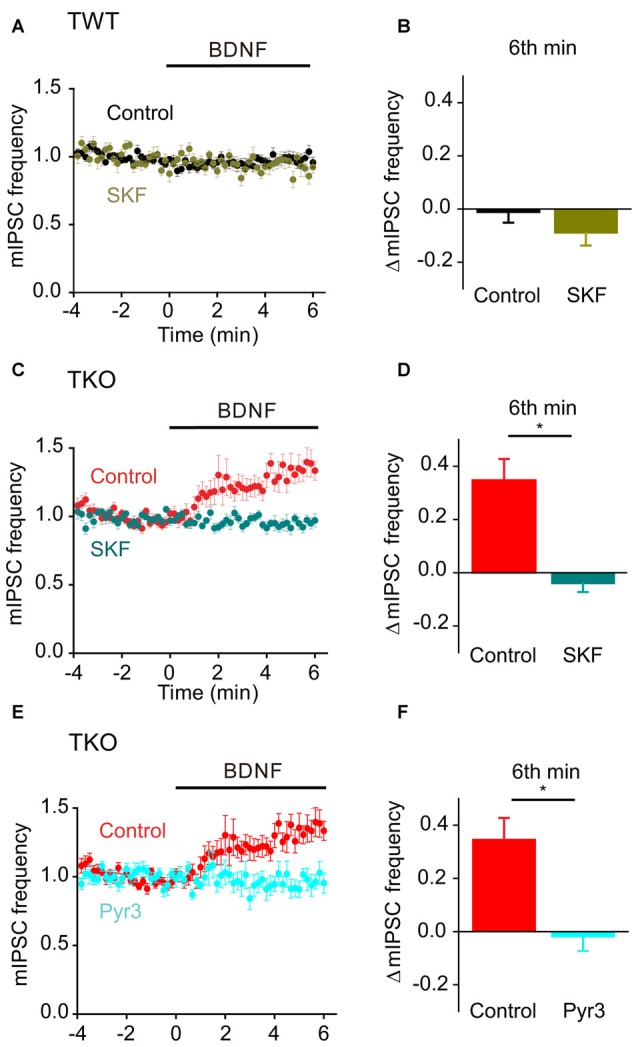
**Role of TRPC channels in presynaptic action of BDNF on spontaneous GABA release in TKO neurons.** **(A)** Time course of changes in normalized mIPSC frequency during BDNF application for TWT neurons without (black, *n* = 21) and with (olive green, *n* = 12) SKF96365 (3 μM). **(B)** Changes in mIPSC frequency during 6th minute of BDNF application in TWT neurons, with and without SKF96365 treatment. **(C)** Time course of changes in normalized mIPSC frequency in TKO neurons during BDNF application without (red, *n* = 18) and with (turquoise, *n* = 12) SKF96365 treatment. **(D)** Changes in mIPSC frequency in TKO neurons during 6th minute of BDNF application, with and without SKF96365. **(E)** Time course of changes in normalized mIPSC frequency in TKO neurons during BDNF application without (red, *n* = 18) and with (sky blue, *n* = 8) Pyr3 treatment (3 μM). **(F)** Changes in mIPSC frequency in TKO neurons during 6th minute of BDNF application, with and without Pyr3.

Finally, we asked whether this BDNF-induced Ca^2+^ influx is carried by TRPC3 channels. BDNF had no effect on mIPSC frequency in TWT neurons treated with SKF96365 (Figures 11A,B, olive). In contrast, SFK96365 completely abolished the ability of BDNF to increase mIPSC frequency in TKO neurons (Figures 11C,D, turquoise). Pyr3 also inhibited the increase in mIPSC frequency caused by BDNF in TKO neurons (Figures 11E,F, cyan). We therefore conclude that the ability of BDNF to increase spontaneous GABA release in TKO neurons is caused by Ca^2+^ influx through TRPC3 channels. This is similar to the case for BDNF regulation of spontaneous glutamate release, except the former is sustained for at least 6 min (Figure 8B), while the latter is transient and lasts for approximately 2 min (Figure 5A).

## Discussion

BDNF regulates synaptic transmission and synaptic plasticity in multiple ways. We have studied one of the most prominent effects of this neurotrophin, the enhancement of spontaneous transmitter release from presynaptic terminals. Our study provides new insights into the intracellular signaling pathways that mediate BDNF action at presynaptic terminals.

### Two Parallel Pathways for BDNF Presynaptic Action

We found that BDNF employs two parallel pathways to increase spontaneous glutamate release and that these pathways mediate responses with different kinetics. Specifically, a TRPC3-mediated Ca^2+^ influx pathway is responsible for an initial, transient increase in mEPSC frequency, while MAPK-mediated phosphorylation of synapsins is required for the sustained enhancement of mEPSC frequency in response to BDNF. These two components together are sufficient to completely account for the ability of BDNF to enhance spontaneous glutamate release. We found that BDNF can also influence spontaneous GABA release via TRPC3-mediated Ca^2+^ influx, though normally this pathway is inhibited by synapsins.

At glutamatergic synapses, there is some temporal overlap between the transient response, which begins almost immediately and subsides within 1–2 min (e.g., Figures 4A, 5A), and the sustained response, which gradually rises from the start of BDNF application and only reaches a peak 2–4 min later (e.g., Figures 2B, 3A,C). This temporal overlap is emphasized when measuring responses after 1 min of BDNF treatment, which is why conditions that eliminate the sustained component have some effect on responses measured at 1 min (Figures 4B, 5B) and why conditions that eliminate the transient component do not completely eliminate the response measured at 1 min (Figures 2C, 3B,D). However, this overlap does not change the conclusion that these two components of BDNF response are distinct and can be differentiated by their transient or sustained characteristics.

### TRPC Channels and the Transient Initial Response to BDNF

Our analysis of the effect of BDNF on spontaneous glutamate release revealed an initial component of BDNF response that is experimentally distinct from the sustained, MAPK- and synapsin-dependent component. The initial component was attenuated by removal of external Ca^2+^ and by treatment with blockers of TRPC3 channels and, thus, is mediated by Ca^2+^ influx through TRPC3 channels. This conclusion is consistent with the results of previous pharmacological experiments (Amaral and Pozzo-Miller, 2012) that did not specify which TRPC isoform was involved. Although BDNF is known to activate several TRPC family members—including TRPC3 (Amaral and Pozzo-Miller, 2007b), TRPC5 (Fortin et al., 2012), and TRPC6 (Zhou et al., 2008) our study has established that the TRPC3 channel mediates BDNF-induced spontaneous glutamate and GABA release. Our results are consistent with a previous report that TRPC3 channels are present in presynaptic terminals (Singh et al., 2004).

Further support for the involvement of a Ca^2+^ influx pathway in the transient initial response to BDNF comes from the observation that the kinetics of this component—as determined in conditions where the other component is abolished, for example in synapsin TKO neurons (Figure 5)—had a time course very similar to that of the transient rise in presynaptic [Ca^2+^]_i_ induced by BDNF treatment (Figure 1). While BDNF previously has been shown to increase [Ca^2+^]_i_ in the soma and dendrites of hippocampal neurons (Amaral and Pozzo-Miller, 2007a,b; Nakata and Nakamura, 2007; Li et al., 2010), ours is the first direct demonstration of a BDNF-induced rise in [Ca^2+^]_i_ within presynaptic terminals. Although the rise in presynaptic [Ca^2+^]_i_ produced by BDNF is modest, on the order of 100 nM, this is quantitatively sufficient to cause the 50% increase in mEPSC frequency observed (Ravin et al., 1999; Angleson and Betz, 2001; Schneggenburger and Rosenmund, 2015). We therefore propose that the initial enhancement of mEPSC frequency by BDNF is caused by the rise in presynaptic [Ca^2+^]_i_ that we observed.

### MAPK and Synapsins Mediate the Sustained Response to BDNF

The MAPK-synapsin pathway has previously been implicated in the presynaptic actions of BDNF (Jovanovic et al., 1996, 2000, 2001; Kao et al., 2017). In addition, this pathway has been shown to regulate both long-term and short-term synaptic plasticity (Jovanovic et al., 2001; Kushner et al., 2005; Chin et al., 2006). A role for synapsins I and II in BDNF-evoked glutamate release was established via measurements of glutamate release from synaptosomes; in these experiments, the response to BDNF was abolished in synaptosomes prepared from the brains of synapsin I/II knockout mice (Jovanovic et al., 2000). Very recently, it was reported that the ability of BDNF to enhance spontaneous glutamate release is abolished in neurons from synapsin I/II/III TKO mice, where the possible contributions of every synapsin isoform can be excluded (Kao et al., 2017). By examining the kinetics of the BDNF response of glutamatergic neurons, we could determine that deletion of all synapsins abolished the sustained enhancement of mEPSC frequency caused by BDNF, consistent with the results of previous experiments. Further, we could discern a smaller, initial component of BDNF response mediated by Ca^2+^ influx.

Because synapsins are well-established substrates of MAPK, we propose that MAPK works upstream of synapsins in this pathway by phosphorylating synapsins (Figure 7). Our main experimental support for this proposal is that application of a MAPK inhibitor, which completely eliminated the sustained response to BDNF in TWT neurons, had no effect in synapsin TKO neurons (Figures 5C,D). However, the synapsin isoforms involved remains to be determined. Among the five main mammalian synapsin isoforms, only synapsins Ia, Ib and IIIa have experimentally determined MAPK phosphorylation sites. Synapsin I isoforms are likely to be important for the presynaptic BDNF response, because BDNF causes synapsin Ia to be phosphorylated at MAPK phosphorylation sites Ser 62, Ser 67 and Ser 549 (Jovanovic et al., 1996) and deletion of the synapsin I gene abolishes the enhancement of glutamate release by BDNF in synaptosomes (Jovanovic et al., 2000). It remains possible that isoforms other than synapsin I may also be involved: the acute enhancement of evoked EPSCs by BDNF is intact in synapsin I KO neurons, as well as in synapsin I KO neurons expressing loss-of-function mutations in synapsin I MAPK phosphorylation sites (S62A, S67A; Valente et al., 2012). Ser 470 in synapsin IIIa has also been determined to be a substrate for MAPK (Porton et al., 2004), though whether this site is phosphorylated in response to BDNF is not yet known. Further work will be needed to identify the key synapsin isoforms, and their phosphorylation sites, involved in mediating the presynaptic effects of BDNF.

The mechanism by which the MAPK/synapsin pathway mediates the enhancement of spontaneous glutamate release by BDNF also is unknown. Conventionally, synapsins are though to maintain glutamatergic synaptic vesicles within a reserve pool (Hilfiker et al., 1999). It is possible that MAPK kinase signaling enhances mobilization of vesicles from this pool, though the main synapsin isoform involved in maintaining vesicles within the reserve pool is synapsin IIa, which is not yet established as a MAPK substrate (Gitler et al., 2008). Other presynaptic mechanisms might also be involved, such as alteration of synaptic vesicle recycling (Chi et al., 2003) or enhancement of short-term synaptic potentiation, a process thought to result from enhancement of the readily-releasable pool of synaptic vesicles (Giachello et al., 2010).

### BDNF Regulation of Spontaneous GABA Release

Previous work has yielded diverse conclusions regarding the acute effects of BDNF at GABAergic presynaptic terminals. While application of BDNF for 15–25 min increases mIPSC frequency (Wardle and Poo, 2003), application of BDNF for 5–10 min reportedly does not increase mIPSC frequency (Colino-Oliveira et al., 2016). Our results are consistent with the latter findings: a 6-min long application of BDNF had no effect on mIPSCs in WT neurons. Thus, the duration of BDNF exposure is an important variable for regulating spontaneous GABA release.

Remarkably, even a 6-min long application of BDNF was sufficient to elevate mIPSC frequency in synapsin TKO neurons. This response to BDNF was mediated by the same TRPC3-dependent Ca^2+^ influx pathway involved in BDNF regulation of spontaneous glutamate release. Presumably the differences in the time course of responses in glutamatergic and GABAergic synapses reflect differences in the gating of TRPC3 channels in the two types of nerve terminals: the upstream intracellular signal transduction (e.g., PLC pathway) mediating TRPC3 opening could be different in the two cases, or perhaps the same signaling pathway is simply operating on different time scales.

Because BDNF responses were not observed at GABAergic synapses of WT mice, we conclude that synapsins serve to prevent BDNF regulation of GABA release via TRPC3 under normal physiological conditions. This is not the only difference in synapsin action between glutamatergic and GABAergic presynaptic terminals: in cultured hippocampal neurons, only synapsin IIa can rescue defects in glutamatergic vesicle reserve pool size observed in TKO neurons (Gitler et al., 2008), while virtually any synapsin isoform can rescue the slowing of GABA release from the readily releasable pool of GABAergic vesicles found in TKO neurons (Song and Augustine, 2016). While we do not understand how synapsins inhibit BDNF signaling in GABAergic terminals, possible mechanisms include Ca^2+^-dependent ATP binding and hydrolysis (Esser et al., 1998; Hosaka and Südhof, 1998a,b, 1999), mitochondrial localization and ATP production (Su et al., 2014), or other unknown enzymatic activity (Kahn and Besterman, 1991; Esser et al., 1998).

In conclusion, our data show that two different signaling pathways are involved in the presynaptic actions of BDNF. For glutamatergic presynaptic terminals, a TRPC3-mediated influx of Ca^2+^ elevates spontaneous glutamate release by elevating presynaptic Ca^2+^ concentration and a second pathway is based on MAPK-mediated phosphorylation of synapsins that causes a sustained enhancement of glutamate release via an unknown mechanism. In contrast, at GABAergic nerve terminals, synapsins opposes the ability of BDNF to enhance Ca^2+^ influx through a TRPC3 channel.

## Author Contributions

QC, S-HS and GJA designed experiments and wrote the article; QC and S-HS performed experiments and analyzed data.

## Conflict of Interest Statement

The authors declare that the research was conducted in the absence of any commercial or financial relationships that could be construed as a potential conflict of interest.
